# 

**Published:** 1893-04

**Authors:** 


### Illinois State Dental Society and Iowa State Dental Society —Joint Meeting.

The twenty-ninth annual meeting of the Illinois State Dental Society will be held at Rock Island, May 9–12, inclusive. The thirtieth annual meeting of the Iowa State Dental Society will be held at Davenport, May 9–12, inclusive. These cities are located on opposite sides of the Mississippi river and arrangements will be made to hold the meeting jointly, so that those in attendance at the meeting of either society will have an opportunity to listen to the papers, take part in the discussions and witness the clinics of both societies. No efforts will be spared to make this union meeting one of the most interesting in the history of each society. Members of both societies are urgently requested to attend. All dentists are cordially invited to be present. Every one should bring models, specimens, appliances or anything that may be of interest to the profession.

LOUIS OTTOFY, Sec'y Illinois State Dental Society, Chicago.

W. O. KULP, Chairman Executive Committee Iowa State Dental Society, Davenport, Iowa.

### Seventh District Dental Society of Ohio.

*To the Members of the Seventh District Dental Society of Ohio:*

After due consultation with the Executive Committee, and also with a number of the members, it was deemed wise to postpone our regular annual meeting—which was to take place here on the third Tuesday in May—for one year. This conclusion was reached on account of the more important meetings which are to be held during the summer, in consequence of which it was impossible to make a satisfactory program.

B. F. JOHNSON, President.

CAMDEN, O., March 25, 1893.

### Kentucky State Dental Society.

The annual meeting of the Kentucky State Dental Association will be held at Richmond, Kentucky, the first Tuesday in June. A number of papers have already been promised. All should consider it a privilege to contribute to make this meeting an interesting and profitable one. All members of the profession are cordially invited to attend.

B. Oscar Doyle.
H. B. Tileston,
(Acting Secretary.)
Jas. H. Letcher,
Executive Committee.

### Editorial.

### The Dental Register,

**A Monthly Magazine Devoted to the Interests of the**

### DENTAL PROFESSION.

*Terms Reduced to $2.00 Per Year. Single Copies, 25 Cents.*

### EDITED BY PROF. J. TAFT, D.D.S.

—AND—

### Published by SAM'L A. CROCKER & CO., Ohio Dental and Surgical Depot.

The volume commences in January and closes in December.

The Register being issued monthly is always fresh, therefore Indispensable to the practitioner who desires to keep posted up with the new inventions and improvements which are daily developed. Neither expense nor effort will be spared to give value and variety to its contents. Articles will be illustrated with well executed engravings whenever they will add to the interest or assist to explanation.

Its extensive circulation and frequency of issue make it one of the BEST DENTAL ADVERTISING MEDIUMS in the United States.

All subscriptions must begin with January or July.

Local or special notices, five lines or less, will be inserted for $3 each insertion; over five lines, 50 cts. per line.

All advertising bills payable in advance, or at furthest, after the issue of the first number containing the advertisement. All transient advertisements to be paid for in advance.

☞ CONTRIBUTIONS SOLICITED.

Exclusively Editorial Communications should be addressed to the Editor.

The latest number of the Register may be ordered with or without other good[illegible] any time, and all letters should be addressed to

